# Household Transmission of *Leptospira* Infection in Urban Slum Communities

**DOI:** 10.1371/journal.pntd.0000154

**Published:** 2008-01-30

**Authors:** Elves A. P. Maciel, Ana Luiza F. de Carvalho, Simone F. Nascimento, Rosan B. de Matos, Edilane L. Gouveia, Mitermayer G. Reis, Albert I. Ko

**Affiliations:** 1 Centro de Pesquisas Gonçalo Moniz, Fundação Oswaldo Cruz, Ministério da Saúde, Salvador, Bahia, Brazil; 2 Municipal Secretary of Health for Salvador, Salvador, Bahia, Brazil; 3 State Secretary of Health for Bahia, Salvador, Bahia, Brazil; 4 Division of International Medicine and Infectious Disease, Weill Medical College of Cornell University, New York, New York, United States of America; Institut Pasteur, France

## Abstract

**Background:**

Leptospirosis, a spirochaetal zoonotic disease, is the cause of epidemics associated with high mortality in urban slum communities. Infection with pathogenic *Leptospira* occurs during environmental exposures and is traditionally associated with occupational risk activities. However, slum inhabitants reside in close proximity to environmental sources of contamination, suggesting that transmission during urban epidemics occurs in the household environment.

**Methods and Findings:**

A survey was performed to determine whether *Leptospira* infection clustered within households located in slum communities in the city of Salvador, Brazil. Hospital-based surveillance identified 89 confirmed cases of leptospirosis during an outbreak. Serum samples were obtained from members of 22 households with index cases of leptospirosis and 52 control households located in the same slum communities. The presence of anti-*Leptospira* agglutinating antibodies was used as a marker for previous infection. In households with index cases, 22 (30%) of 74 members had anti-*Leptospira* antibodies, whereas 16 (8%) of 195 members from control households had anti-*Leptospira* antibodies. Highest titres were directed against *L. interrogans* serovars of the Icterohaemorrhagiae serogroup in 95% and 100% of the subjects with agglutinating antibodies from case and control households, respectively. Residence in a household with an index case of leptospirosis was associated with increased risk (OR 5.29, 95% CI 2.13–13.12) of having had a *Leptospira* infection. Increased infection risk was found for all age groups who resided in a household with an index case, including children <15 years of age (P = 0.008).

**Conclusions:**

This study identified significant household clustering of *Leptospira* infection in slum communities where recurrent epidemics of leptospirosis occur. The findings support the hypothesis that the household environment is an important transmission determinant in the urban slum setting. Prevention therefore needs to target sources of contamination and risk activities which occur in the places where slum inhabitants reside.

## Author Summary

Leptospirosis has emerged to become an urban slum health problem. Epidemics of severe leptospirosis, characterized by jaundice, acute renal failure and haemorrhage, are now reported in cities throughout the developing world due to rapid expansion of slum settlements, which in turn has produced the ecological conditions for rodent-borne transmission of the spirochete pathogen. A survey was performed in the city of Salvador, Brazil, to determine whether the risk of *Leptospira* infection clustered in households within slum communities in which a member had developed severe leptospirosis. We found that members of households with an index case of leptospirosis had more than five times the risk of having serologic evidence for a prior infection than members of neighbourhood households in the same communities. Increased risk of infection was found among all age groups who resided in these households. The finding that *Leptospira* infection clusters in specific slum households indicates that the factors associated with this environment are important determinants for transmission. Further research is needed to identify the sources of contamination and risk exposures which occur in the places where slum inhabitants reside such that effective community-based prevention of urban leptospirosis can be implemented.

## Introduction

Leptospirosis is an important zoonotic health problem because of its life-threatening clinical manifestations, Weil's disease and severe pulmonary haemorrhage syndrome, for which fatality is 10 to 50% [1]. Moreover there has been growing awareness of the large under-recognized disease burden that leptospirosis imparts in developing countries [2]. Leptospirosis is an environmentally-transmitted disease. Pathogenic spirochetes of the genus *Leptospira* establish chronic carriage in the kidney tubules of wild and domestic mammalian reservoirs and persist for weeks in the environment after excretion from the host [3]. The major mode of transmission to humans is indirect contact with water or moist soil contaminated with the urine of animal reservoirs [3]. Leptospirosis is associated with a spectrum of environmental settings and risk exposures. Recreation, travel and water sports have become significant risk factors in industrialized countries [3],[4],[5], as exemplified by outbreaks during triathlon and adventure tourisms events [6],[7]. In developing countries situated in tropical climates, leptospirosis is an endemic disease of rural-based populations engaged in subsistence farming, sharecropping and animal husbandry [3],[8].

Furthermore leptospirosis has emerged to become an urban slum health problem in developing countries [9],[10]. The rapid expansion of slum settlements, in which 1 billion of the world's population reside [11], has produced the ecological conditions for rodent-borne transmission [9],[12]. Epidemics of severe leptospirosis are now reported in cities throughout the developing world [1]. In Brazil alone, more than 10,000 cases of severe leptospirosis are reported each year [1] during outbreaks that occur in major urban cities [9],[13],[14],[15],[16]. During these outbreaks, leptospirosis cases cluster in slum settlements which lack adequate sewage systems and refuse collection services [9],[13],[17].

Public health responses to urban leptospirosis require an improved understanding of the specific exposures in slum communities which lead to epidemic transmission. Urban outbreaks are associated with heavy seasonal rainfall and flooding [9],[13],[16],[18]. Environmental surface waters in slum communities, as found in a study in Peru [12], contain high concentrations of pathogenic *Leptospira* serovars which are associated with acquiring severe disease forms. Leptospirosis is traditionally considered an occupational disease, since work-related activities are frequently identified as risk exposures [3]. However slum inhabitants reside in close proximity to environmental sources of contamination, such as open sewers, flood areas and trash collections [19]. Determining whether transmission occurs in the household environment will be essential to designing and implementing effective community-based interventions for which at present, none are available.

In Salvador, a city of 2.4 million inhabitants in Northeast Brazil, outbreaks of leptospirosis occur annually during the seasonal period of heavy rainfall [9],[19]. A case-control investigation found that residence in proximity of open sewers and peridomicilary sighting of rats to be risk factors for acquiring severe leptospirosis [19], suggesting a role for household-related environmental exposures in transmission. In this study, we surveyed members of households in which an index case of severe leptospirosis resided and control households that were situated in the same slum communities. A serologic evaluation was performed of samples obtained from this survey to determine whether the risk for *Leptospira* infection clustered within households.

## Methods

### Identification of index cases of severe leptospirosis

Active surveillance consecutively identified patients that fulfilled a clinical case definition for severe leptospirosis [9],[19] and were admitted to the infectious disease hospital during an outbreak that occurred in Salvador, Brazil in 2001. According to health secretary protocols, suspected cases of leptospirosis from the metropolitan region are referred to this site. Acute and convalescent-phase serum samples were collected and evaluated in the microscopic agglutination test (MAT) according to protocols previously described [9]. A laboratory-confirmed case of leptospirosis was defined as the presence of a four-fold rise in the MAT titre between paired acute and convalescent-phase serum samples or a titre of >1∶800 in a single sample [9].

### Community sites and household survey

A survey was performed between May and October 2001 of members of case households, in which index cases of confirmed leptospirosis resided at the time of illness, and neighbourhood control households selected from the same slum communities. The study team visited the residences of the first 22 cases that were identified to have a confirmed diagnosis of leptospirosis. Cases resided in 19 densely-populated slum neighbourhoods, most of which were situated in the periphery of Salvador. Although the majority (>90%) of the households have access to potable water in these communities, more than 30% are served by open sewage systems. Study communities were built on poor land quality which are at risk for flooding during the seasonal period of heavy rainfall. Due to the lack of refuse collection services, refuse accumulates in open deposits and open sewers. Dogs, cats and chickens are domestic animals which are encountered in households and the peridomicilary environment. Due to the poor overall sanitation infrastructure, the environment surrounding households in these communities is infested with domestic rats, in particular *Rattus norvegicus*.

Neighbourhood control households were selected according to the sampling scheme used in a previous case-control investigation [19]. Households were surveyed which were located a distance of five domiciles from the case household, and at every household thereafter, until a neighbourhood control household was identified which did not have a member who was diagnosed at a health care facility as having leptospirosis in 2001 and agreed to participate in the study. This strategy was chosen to avoid sampling control subjects who resided in close proximity to households and may therefore have had shared risk exposures. Subjects were interviewed to determine whether they had a history of a recent diagnosis of leptospirosis. In addition, we screened the database of cases identified during active hospital-based surveillance to exclude the possibility that subjects were recent leptospirosis patients. A total of four control households were selected for each of the first four identified case households by sampling domiciles in perpendicular directions. Two control households were selected for each of the remaining 18 case households by sampling domiciles in opposite directions.

Eligible subjects were defined as those who were greater than five years of age and resided in case and control households for more than one month prior to identification of the index case of leptospirosis. The study team identified and recruited subjects from case households and their respective control households during the same community site visit. All subjects were enrolled into the study according to written informed consent protocols approved by the Ethical Committee in Research of the Oswaldo Cruz Foundation and IRB Committee of Weill Medical College of Cornell University. The study team performed interviews to obtain demographic information and obtained blood samples from which sera was separated and stored for serologic analyses.

### Serologic evaluation

The MAT was performed to determine the presence of anti-*Leptospira* antibodies in subject samples [20] and used a panel that included seven pathogenic reference strains, *L. interrogans* serovars Autumnalis, Canicola, Copenhageni, Icterohaemorrhagiae and Saxkoebing, *L. kirschneri* serovar Grippotyphosa, and *L. borgpetersenii* serovar Ballum, one non-pathogenic reference strain, *L. biflexi* serovar Patoc (WHO/FAO Collaborating Centre for Reference and Research on Leptospirosis, Royal Tropical Institute, Holland), and two clinical isolates of *L. interrogans* serovars Canicola and Copenhageni [9]. The use of the reduced panel of ten strains in MAT evaluations had the same predictive value in identifying positive agglutination reactions among confirmed leptospirosis cases in Salvador [9] as did the WHO recommended battery of 18 reference serovars [20]. Operators were blinded to whether samples were from case and control household members. Each serum sample was tested at three dilutions, 1∶25, 1∶50 and 1∶100, and when agglutination was observed at the 1∶100 dilution, was titrated to determine the highest agglutination titre.

### Statistical analysis

Epidemiological and laboratory information for subjects were entered into an EpiInfo version 6.04 software system (Centres for Diseases Control and Prevention) database. Chi-square and Wilcoxon rank sum tests were used to compare categorical and continuous data, respectively, for eligible subjects who were and were not enrolled in the study and members of case and control households and to evaluate differences between highest agglutination titres for members of case and control households. A P value of less than 0.05 in two sided testing was used as criteria for a statistically significant difference. Analyses evaluated a range of reciprocal MAT titres against pathogenic serovars as threshold criteria for a prior *Leptospira* infection. The presumptive infecting serovar was defined as the pathogenic serovar against which the highest agglutination titre was directed. The chi-square or Fisher's exact tests were used to evaluate for significant associations between residence in a household with an index case of leptospirosis and the risk for acquiring anti-*Leptospira* antibodies. The CSAMPLE program of the EpiInfo version 6.04 was used to obtain estimates for the OR and 95% CI which were adjusted for the sampling design effect and weighted for the number of eligible household members. Serologic results from index cases of leptospirosis were excluded from the analyses.

## Results

Active surveillance detected an outbreak of severe leptospirosis in Salvador during the seasonal period of heavy rainfall in 2001. Between March and October, 124 suspected cases were identified which resided within the city whereas 16 cases were identified during the preceding four month period. Leptospirosis cases were residents of 70 slum neighbourhoods (*bairros*) situated within the city. Cases were mostly adults (mean age±standard deviation, 35.2±13.5 years) and males (86% of 124 cases) and hospitalized with manifestations of Weil's disease such as jaundice (77% of 124 cases) and acute renal failure (73% with serum creatinine >2.0 mg/dL). Overall case fatality was 10% (12 of 124 deaths). Among the 124 suspected cases, 89 (72%) had a laboratory-confirmed diagnosis of leptospirosis.

A survey was performed of 22 case households in which an index case of confirmed leptospirosis resided at the time of the outbreak and 52 control households situated in the same slum communities as case households. Households were enrolled from 19 (27%) of the 70 slum neighbourhoods in which leptospirosis cases were identified during the outbreak. Among 79 and 229 eligible subjects who resided in respectively, case and control households, 74 (94% of 79) and 195 (85% of 229) subjects were enrolled into the study. Subjects were healthy at the time of identification and did not report symptoms or signs of leptospirosis in the previous one-year period. One member of an index case household was previously hospitalized for leptospirosis. Neighbourhood control households were located between 15 and 45 meters from their respective index case household. Case and control households did not differ with respect to number of household members (median 3 vs. 3) and median monthly income (US$64 vs. 84) of the head-of-the-household. Subjects from cases and control households were similar with respect to median age (22 vs. 26 years, respectively) and gender (males, 40 vs. 42%).

Table 1 shows the distribution of highest agglutination titres against pathogenic Leptospira serovars among subjects. Members of case households had significantly higher agglutination titres than those from control households located in the same slum communities (Wilcoxon rank sum test, P<0.001). Among members of case households, 18%, 23% and 30% had MAT titres ≥1∶100, 1∶50, and 1∶25, respectively, against a pathogenic serovar (Table 2). Among members of control households, 5%, 7% and 8% had titres ≥1∶100, 1∶50, and 1∶25, respectively. Across the range of highest MAT titres, the majority of agglutination reactions recognized *L. interrogans* serovars of serogroup Icterohaemorrhagiae, serovars Copenhageni and Icterohaemorrhagiae (Table 1). Among members of case and control households with MAT titres ≥1∶25, 95% (21 of 22 subjects) and 100% (16 of 16 subjects), respectively, were directed against serovars Copenhageni and Icterohaemorrhagiae.

**Table 1:** Distribution of microscopic agglutination titres for members of households with an index case of severe leptospirosis and neighbourhood control households from slum communities in Salvador, Brazil.

	No. with highest agglutination titres							
*Leptospira* Serovars	Negative	1∶25	1∶50	1∶100	1∶200	1∶400	1∶800	1∶1600
**Members of households with index cases of leptospirosis (N = 74)** a								
*L. interrogans* serogroup Icterohaemorrhagiae	-	5	3	11	0	1	0	0
serovar Copenhageni	-	4	2	7	0	1	0	0
serovar Icterohaemorrhagiae	-	0	1	2	0	0	0	0
Both^b^	-	1	0	2	0	0	0	0
*L. interrogans* serovar Saxkoebing	-	0	1	0	0	0	0	0
Total (% subjects)^c^	52 (70)	5 (7)	4 (5)	11 (15)	0 (0)	1 (1)	0 (0)	1 (1)
**Members of neighbourhood control households (N = 195)** d								
*L. interrogans* serogroup Icterohaemorrhagiae	-	2	4	5	2	3	0	0
serovar Copenhageni	-	1	3	4	2	3	0	0
serovar Icterohaemorrhagiae	-	0	1	0	0	0	0	0
Both^b^	-	1	0	1	0	0	0	0
Total (% subjects)^c^	179 (92)	2 (1)	4 (2)	5 (3)	2 (1)	3 (2)	0 (0)	0 (0)

a Highest titres did not react against *L. interrogans* serovars Autumnalis and Canicola, *L. kirschneri* serovar Grippotyphosa, and *L. borgpetersenii* serovar Ballum.

b Highest titres recognized *L. interrogans* serovars Copenhageni and Icterohaemorrhagiae.

c Total percentages do not equal 100% because of rounding.

d Highest titres did not react against *L. interrogans* serovars Autumnalis, Canicola and Saxkoebing, *L. kirschneri* serovar Grippotyphosa, and *L. borgpetersenii* serovar Ballum.

**Table 2:** Prevalence of anti-*Leptospira* antibodies among members of households with an index case of severe leptospirosis and neighbourhood control households.

Reciprocal MAT titre	Households with index cases (n = 74)	Neighbourhood control households (n = 195)	OR (95% CI)^a^
	No. (%)		
Against pathogenic serovars^b^			
≥25	22 (30)	16 (8)	5.29 (2.13–13.12)
≥50	17 (23)	14 (7)	3.71 (1.47–9.32)
≥100	13 (18)	10 (5)	4.42 (1.53–12.76)
Against nonpathogenic serovar^c^			
≥25	11 (15)	40 (21)	0.76 (0.29–1.98)
≥50	5 (7)	4 (3)	2.65 (0.27–25.83)
≥100	1 (2)	1 (1)	2.29 (0.14–36.96)

a MAT, microscopic agglutination test.

b OR and 95%CI were adjusted for the design effect associated with sampling households in the survey.

c *Leptospira biflexa* serovar Patoc.

Members of case households were 4.42 times (95% CI 1.53–12.76) more likely than members of control households to have agglutinating antibodies against a pathogenic *Leptospira* serovar with an titre of ≥1∶100 (Table 2), a criteria commonly used to define probable infection in clinical cases of leptospirosis [21]. However, significant risk associations were found among members of case households when lower titres were used as threshold values for prior infection (OR 5.29 and 3.71 for ≥1∶25 and 1∶50, respectively, P<0.05). This risk association was specifically found when agglutinating antibodies against a pathogenic serovar were used as the outcome marker for prior *Leptospira* infection. The prevalence of agglutinating antibodies against a non-pathogenic serovar, *L. biflexi* serovar Patoc, did not differ between members of case and control households, irrespective of the titre used to define a positive result (Table 1).

Within households with index cases of leptospirosis, children and young adults had significantly increased risk of having anti-*Leptospira* antibodies (Table 3). Significantly higher proportions of children in index case households had anti-*Leptospira* antibodies, as defined by a MAT titre ≥1∶25, than children in control households (19 vs. 0%, P = 0.008). Older adults with ≥55 years of age and adults with 15–34 years of age had as well a significantly increased risk for acquiring anti-*Leptospira* antibodies (OR 5.60 and 4.87, respectively, P<0.05) while adults with 35–54 years of age had a increased, albeit non-significant risk (OR 3.18, P = 0.32).

**Table 3:** Prevalence of anti-*Leptospira* antibodies[Table-fn nt108] among age groups residing in households with an index cases of severe leptospirosis and neighbourhood control households.

	Households with index cases		Neighbourhood control households		
Age group	No.	No. positive (%)	No.	No. positive (%)	OR (95% CI)^a^
5–14 years	21	4 (19)	45	0 (0)	P = 0.008
15–34 years	31	12 (39)	94	11 (12)	4.87 (1.73–13.69)
35–54 years	14	3 (21)	38	3 (8)	3.18 (0.75–13.48)
≥55 years	8	3 (38)	18	2 (11)	5.60 (1.70–18.46)

Prevalence of anti-*Leptospira* antibodies is defined as having microscopic agglutination titres ≥1∶25 to a pathogenic *Leptospira* serovar.

a OR and 95%CI were adjusted for the design effect associated with sampling households in the survey.

## Discussion

This study, performed in an endemic region for urban leptospirosis, identified household clustering of *Leptospira* infection within slum communities. Among members who resided in the same household as an index case of leptospirosis, 30% had evidence of a previous infection as determined by the presence of anti-*Leptospira* antibodies in the MAT. These individuals had more than five times the risk for acquiring anti-*Leptospira* antibodies as compared with members of neighbouring households. Household clustering of *Leptospira* infection has not been found in surveys which evaluated for this phenomenon in rural endemic settings [22]. A previous serologic survey, which was also performed in Salvador and used IgM ELISA to detect anti-*Leptospira* antibodies, found that high (41%) proportions of children had antibodies among those residing in households with index cases of leptospirosis [23]. However, a control group was not evaluated in order to assess whether individuals with anti-*Leptospira* antibodies specifically aggregated in index case households. This study, as far as we are aware, is the first to describe household clustering of *Leptospira* infection.

We used the presence of anti-*Leptospira* agglutinating antibodies as a marker of previous infection. The MAT is the standard assay to measure seroprevalence [3],[20]. Although a MAT titre ≥1∶100 is often employed [3], the performance of different MAT titre criteria have not been systematically evaluated in community-based investigations and a consensus does not exist with respect to a standard cutoff titre. In this study, we found that subjects from case households had significantly increased risk (OR 4.42, 95% CI 1.53–12.76) of having an agglutination titre ≥1∶100 against a pathogenic *Leptospira* serovar than subjects from control households. We evaluated lower cut-off criteria of titres ≥1∶50 and ≥1∶25 and found that the use of these criteria predicted the same epidemiological relationship as did the criterion of a titre ≥1∶100 (Table 2). The observed household clustering of individuals with anti-*Leptospira* antibodies does not therefore appear to be an artefact of serologic criteria used.

These findings also indicate that the use of lower cutoff titres (i.e., 1∶25) is a more sensitive yet still specific serologic criterion for a previous *Leptospira* infection in our setting. Isolation studies showed that urban leptospirosis in Salvador is due to the transmission of a single serovar, *L. interrogans* serovar Copenhageni [9],[24]. In this study, more than 95% of subjects with positive MAT titres had agglutinating antibodies which specifically reacted against serovars Copenhageni and Icterohaemorrhagiae. This phenomenon was observed across the range of titre values, including titres of 1∶25 (Table 1). Individuals infected with serovar Copenhageni are expected to have serological cross-reactions against serovar Icterohaemorrhagiae since the both serovars belong to same serogroup [8]. Investigations performed in endemic areas in which several serogroups are circulating found that the predictive value of serology is low with respect to identifying the infecting serogroup [25],[26]. Our findings suggest that in a site where a single agent, serovar Copenhageni, is circulating, previously-infected individuals have agglutination titres which, even when low titres are encountered, are specifically directed against this serovar and serovars from the same serogroup. Non-pathogenic serovars, such as *L. biflexi* serovar Patoc, have been used in MAT strain panels to detect potential cross-reactive antibodies in individuals previously infected with a pathogenic serovar. We found that the titres against non-pathogenic serovars among household members were not significantly associated with residence in a household with an index case of leptospirosis (Table 2), indicating that antibodies that agglutinate non-pathogenic serovars is not be a specific marker for a previous *Leptospira* infection in our setting and may have been induced by unrelated exposures. Together these findings emphasize the need to validate MAT criteria for the specific epidemiological situation in which seroprevalence surveys are being performed.

The study selected households based on identification of an index case of severe leptospirosis. Therefore risk estimates observed in this study may not pertain to households in which a member acquired mild leptospirosis. Furthermore previous infection among subjects occurred over an extended time period since agglutinating antibodies may persist for more than five years [27],[28]. It is therefore likely that severe leptospirosis occurred in households in which a significant proportion of the members had already been exposed to *Leptospira*. Infection among individuals with anti-*Leptospira* antibodies was likely to have been mild or asymptomatic since almost all subjects did not report a history of having leptospirosis. These findings are consistent with those observed in other studies which found asymptomatic infection to be common in endemic areas [29],[30]. Migration may have influenced the estimates of the risk associations. In a cohort investigation of residents from one slum community in Salvador, we found that the annual out-migration rate is approximately 12% (unpublished data). Reliable estimation of the risk attributable to the household will therefore require prospective evaluation of infection and severe disease outcomes in slum community settings.

However, the strength of the association observed for household clustering of *Leptospira* infection risk indicates that household factors play an important role in transmission of leptospirosis in the urban slum setting. Members who reside in the same household may have had similar risk exposures outside the household environment. This explanation may be less likely since children who resided in households with index cases had significantly increased risk for acquiring anti-*Leptospira* antibodies in comparison to neighbourhood control subjects of the same age group (Table 2). Host susceptibility determinants shared among household members are a possible explanation for the observed clustering of individuals with anti-*Leptospira* antibodies. HLA gene polymorphisms have been reported to be associated with the risk of acquiring leptospirosis during an outbreak associated with a triathlon event [31].

Alternatively, the observed clustering of infection risk may reflect shared exposures among household members to transmission sources located in the environment where they reside. Ecological studies [9],[13],[17] and case-control investigations [19] of urban leptospirosis found environmental attributes of slum households to be risk factors for acquiring severe leptospirosis. In these studies, two of which were performed in the same slum communities in Salvador which were investigated in this study [9],[13],[17],[19], risk factors were related to open sewers, poor rainwater drainage systems and associated flooding, lack of refuse collection services, and sighting of rats in the household environment. These environmental sources of contamination are not uniformly distributed in slum settlements and may have contributed to the observed household clustering of infection risk. In addition, our findings suggest that infection risk varies over short distances within these communities. Members of households with index cases of leptospirosis had significantly increased risk of having a previous *Leptospira* infection than their neighbours who resided a distance of 15 to 45 meters from case households. These risk differences may relate to differences between households with respect to rodent population densities and proximity to environmental sources of contamination, such as open sewers, open refuse deposits and flood risk areas. Molecular tools have been recently developed to detect pathogenic *Leptospira* in environmental samples [12],[32],[33] and used to identify that surface waters in slum communities contain high concentrations of pathogenic *Leptospira* which are agents for severe leptospirosis [12]. The use of these tools may enable more refined analyses aimed at identifying specific sources of contamination in the household environment which promote transmission of urban leptospirosis.

Although our findings indicate that household factors are determinants of *Leptospira* transmission in slum communities, work-related exposures may contribute as well to infection in this population. A previous case-control study found workplace-related exposures to contaminated environment to be a risk factor for severe leptospirosis in addition to attributes associated with the household environment [19]. We did not evaluate infection risk at the workplace in this study. Occupations may have been shared among household members and have been a possible confounding factor. Slum residents often engage in informal work, such as small-scale construction and food preparation for vending, in the same environment in which they reside. Targeting exposures in the household environment may therefore have a potential beneficial effect in reducing potential work-related exposures. The study findings may not be generalizable to other epidemiological situations such as rural leptospirosis. Furthermore, the findings do not apply to urban leptospirosis in industrialised countries, which is a sporadic disease associated with inner city homeless populations [34]. However, the finding that leptospirosis is transmitted in the household environment will likely be relevant to the large slum population residing in developing countries which are subjected to similar conditions of poverty and social marginalization.

In conclusion, this study identified significant household clustering of *Leptospira* infection in slum communities, indicating that the household environment and related factors are important determinants for transmission of urban leptospirosis. These findings need to be confirmed in prospective studies of *Leptospira* infection in slum communities. Research is needed to determine whether specific attributes of the slum household environment, such as open sewers, flooding, open refuse deposits, serve as transmission sources and evaluate the role of rats, dogs and other urban reservoirs which are commonly encountered in the places where slum residents reside. Elucidation of the risk factors in slum settlements may lead to targeted community-based interventions for leptospirosis. Moreover, efforts need to be made to raise awareness among slum residents of the risks that occur in their household environment. Implementation of effective community-based interventions will require further studies aimed at identifying the specific activities in the household setting which place slum residents at risk for leptospirosis and developing health education strategies to prevent these risks.

## Supporting Information

Alternative Language Abstract S1 Abstract translated into Portuguese.
(0.03 MB DOC)
