# Supplementary material for: Household Transmission of Leptospira Infection in Urban Slum Communities
Source: PLoS Negl Trop Dis. 2008 Jan 30;2(1):e154. doi: 10.1371/journal.pntd.0000154 (PMC2270796; doi:10.1371/journal.pntd.0000154)
Supplement: Alternative Language Abstract S1 — Abstract translated into Portuguese. (0.03 MB DOC) [file pntd.0000154.s001.doc]

**Resumo**

***Introdução:*** A leptospirose, uma zoonose causada por espiroquetas, é responsável por epidemias com alta letalidade, em favelas urbanas. A infecção com *Leptospira* patogênica ocorre durante exposições ambientais e está tradicionalmente associada com atividades ocupacionais de risco. No entanto, os moradores das favelas residem próximos as fontes ambientais de contaminação, sugerindo que a transmissão durante as epidemias urbanas ocorre no ambiente peridomiciliar.

***Métodos e Resultados*:** Um estudo foi realizado para determinar se a infecção por *Leptospira* estava aglomerada dentro dos domicílios localizados em favelas na cidade de Salvador, Brasil. A vigilância hospitalar identificou 89 casos confirmados de leptospirose durante um surto. Amostras de soro foram obtidas dos moradores de 22 domicílios com um caso índice de leptospirose e dos moradores de 52 domicílios controle localizados nas mesmas comunidades. A presença de anticorpos aglutinantes anti-*Leptospira* foi utilizada como um marcador de infecção prévia. Nos domicílios com um caso índice, 22 (30%) dos 74 moradores tiveram anticorpos anti-*Leptospira*, enquanto 16 (8%) dos 195 moradores dos domicílios controle tiveram anticorpos anti-*Leptospira*. Os títulos mais altos foram dirigidos contra os sorovares de *L. interrogans* do sorogrupo Icterohaemorrhagiae em 95% and 100% dos moradores que apresentavam anticorpos aglutinantes, nos domicílios dos casos e dos controles respectivamente. Residir em um domicílio com um caso índice de leptospirose foi associado com um risco elevado (OR 5,29, 95% CI 2,13-13,12) de infecção prévia por *Leptospira*. O risco aumentado de infecção foi encontrado em todas as faixas etárias residentes no domicilio do caso índice, incluindo crianças com menos do que 15 anos de idade (P=0.008).

***Conclusões*:** Este estudo evidenciou uma aglomeração significativa de infecção por *Leptospira* em favelas onde ocorrem epidemias recorrentes de leptospirose. Estes achados dão suporte para hipótese que o ambiente peridomiciliar seja um determinante importante da transmissão no cenário das favelas urbanas. Portanto, a prevenção precisa ser direcionada para fontes de contaminação e atividade de risco que ocorrem nos locais de residência dos moradores das favelas urbanas.
